# A de novo *SALL4* mutation causes unilateral renal agenesis by misregulating genes involved in kidney development

**DOI:** 10.1186/s13023-025-03833-x

**Published:** 2025-06-07

**Authors:** Rong Zhao, Yali Fan, Jieyan Li, Lin Li, Chenghong Yin

**Affiliations:** 1 Department of Obstetrics, Beijing Obstetrics and Gynecology Hospital, Capital Medical University, Beijing Maternal and Child Health Care Hospital, Chaoyang, Beijing, 100026 China; 2 Central Laboratory, Beijing Obstetrics and Gynecology Hospital, Capital Medical University, Beijing Maternal and Child Health Care Hospital, Dongcheng, Beijing, 100006 China

**Keywords:** Renal agenesis, SALL4, Fetal kidney ureteric bud cells, WNT11, PAX2

## Abstract

**Background:**

SALL4 is a transcription factor that plays a crucial role in early embryonic development and organogenesis, particularly in kidney development, although its specific regulatory mechanisms remain unclear.

**Methods:**

We performed whole-exome sequencing (WES) to identify pathogenic variants in a fetus with unilateral renal agenesis and confirmed a variant in *SALL4* using Sanger sequencing. The expression of wild-type or mutant SALL4 proteins in cells was used to determine whether the level and localization of the proteins were altered by the *SALL4* variant. RNA sequencing was used to identify differentially expressed genes at the transcriptome level due to the SALL4 mutant protein. Finally, key differentially expressed proteins were verified using quantitative PCR and western blotting.

**Results:**

A novel truncating mutation in *SALL4* was identified through WES in a fetus with unilateral renal agenesis. Expression of the truncated SALL4 protein in cells revealed its predominant cytoplasmic localization, unlike the wild-type SALL4 protein, which was localized to the nucleus. Further RNA sequencing analysis indicated that the mutant SALL4 protein lost its transcriptional activation ability, with 1047 genes markedly downregulated compared to cells expressing wild-type SALL4. These downregulated genes were primarily enriched in biological processes such as cell activation, salt transmembrane transporter activity, and calcium ion binding. Additionally, we found that these differentially expressed genes mainly regulated fetal ureteric bud cells, suggesting that SALL4 mutations may ultimately lead to unilateral renal agenesis by affecting the development and function of fetal ureteric bud cells. Among these genes, two proteins crucial for kidney development, WNT11 and PAX2, were significantly downregulated in cells expressing the truncated SALL4 protein, suggesting that WNT11 and PAX2 may mediate the regulatory role of SALL4 in kidney development.

**Conclusion:**

This study elucidated the molecular mechanism by which *SALL4* mutations lead to renal agenesis.

**Supplementary Information:**

The online version contains supplementary material available at 10.1186/s13023-025-03833-x.

## Introduction

*SALL4* encodes a C2H2 zinc-finger transcription factor that plays an important role in the development of various organs. In mouse embryonic stem cells (ESCs), Sall4 prevents precocious activation of neural gene expression but is dispensable for the maintenance of mouse ESC pluripotency [1]. Deletion of *Sall4* leads to embryonic lethality during peri-implantation, suggesting that Sall4 plays an essential role during early embryonic development [2, 3]. Sall4 also regulates the development of neurons, limbs, kidneys, the heart, and female and male germ cells [2, 4–9].

*SALL4* heterozygous mutations can cause Okihiro syndrome, an autosomal dominant inherited disorder called Duane-radial ray syndrome (DRRS, MIM: 607,323) [10]. Clinical manifestations include Duane anomaly, radial ray malformation that includes hypoplasia or aplasia of the thumbs and/or radii, shortened forearms, and duplication of the thumb. Patients with DRRS also have other symptoms, including abnormal ear shape, hearing loss, congenital heart anomalies, renal abnormalities, choanal atresia, and short stature. In addition, studies have reported that mutations in the *SALL4* gene can lead to non-DRRS-related diseases, such as premature ovarian insufficiency [11] and kidney abnormalities, including renal agenesis [12].

At the molecular level, previous studies have found that TBX5 can activate the expression of SALL4 [13], which in turn forms heterodimers with SALL1 [2]. The three mesodermal proteins, TBX5, SALL4, and SALL1, are involved in the development of the radii, heart, and kidneys. This loop is completed by the stimulation of the WNT signaling pathway through SALL1, further enhancing *SALL4* expression through the binding of LFF1/TCF to the *SALL4* promoter region [14]. Therefore, *SALL4* mutations can affect mesoderm development, leading to the abnormal development of the heart, kidneys, and spine [15].

Here, we report a fetus with absence of the radius, renal agenesis, right kidney duplication, and a single umbilical vein detected through ultrasound. WES was performed on the fetus and a frameshift variant in *SALL4* was identified. This variant resulted in the truncation of the SALL4 protein. By expressing *SALL4* wild-type plasmids or truncated mutant plasmids in cell lines, we found that, compared to the SALL4 wild-type expression group, the truncated mutant protein expression group had 1047 downregulated genes. These genes were mainly enriched in the fetal kidney ureteric bud cells, indicating that renal agenesis caused by *SALL4* variant may be related to developmental abnormalities during the fetal kidney ureteric bud cell period. In addition, we found that SALL4 regulated the expression of important genes related to kidney development, such as *WNT11* and *PAX2*.

## Methods

### Patients

A 35-year-old pregnant woman was recruited at Beijing Obstetrics and Gynecology Hospital, Capital Medical University. The patient was primiparous and conceived naturally. At 12 weeks of pregnancy, ultrasound suggested that the nuchal translucency (NT) was 1.8 mm. Although the pregnant woman was of advanced maternal age, she refused amniocentesis and requested non-invasive testing. Non-invasive DNA testing showed a low risk. Ultrasound examination revealed fetal aplasia of the radii, left renal agenesis, right kidney duplication, and a single umbilical artery. The pregnant woman was admitted for diagnostic amniocentesis and labor induction with Rivanol amniocentesis. She had an uneventful miscarriage and was discharged after three days. There were no obvious abnormalities in the macroscopic appearance of the aborted fetus. The autopsy report of the miscarried fetus revealed left renal agenesis, gallbladder agenesis, abnormal development of the hands and feet, internal rotation of the hands by 180°, and internal rotation of the feet. The structure of the cortex and medulla of the right kidney was clear, and the structure of the glomeruli and their affiliated nephrons was also clear.

This study was approved by the Ethics Committee of the Beijing Obstetrics and Gynecology Hospital, Capital Medical University (2018-KY-003-01). Written informed consent was obtained from the pregnant woman. A peripheral blood sample of 5 mL was collected.

### WES and Sanger sequencing validation

Genomic DNA was extracted from the amniotic fluid samples. WES was performed at the Shenzhen Huada Medical Laboratory. First, the DNA library was prepared. DNA from the exons and adjacent splicing regions was captured and enriched using KAPA HyperExome probes (KAPA Biosystems, Inc., Wilmington, MA, USA). Finally, high-throughput sequencing was performed using the MGISEQ-2000 sequencing platform. The quality control standard for the sequencing data was as follows: the average sequencing depth of the target area was ≥ 180×, and the proportion of sites with an average sequencing depth of > 20× in the target area was > 95%. Sequence reads were aligned to the UCSC hg19 human reference genome using the Burrows-Wheeler Alignment (BWA) tool. Variants with allele frequencies of > 1% in the databases were excluded. The databases included the 1000 Genomes (1000G, http://browser.1000genomes.org/index.html), Single Nucleotide Polymorphism Database (dbSNP, http://www.ncbi.nlm.nih.gov/snp/), NHLBI Exome Sequencing Project Database (ESP6500, http://evs.gs.washington.edu/EVS/), and Genome Aggregation Database (gnomAD, https://gnomad.broadinstitute.org). Sanger sequencing was used to validate the variant in the fetus, pregnant woman, and her husband.

### Vector construction, cell culture, and plasmid transfection

The full-length *SALL4* (NM_020436), with a C-terminal 3 × Flag sequence in the pcDNA3.1 vector, was purchased from YouBio (F122676, China). The DNA sequence corresponding to the SALL4 truncated protein p.Thr182* was cloned into the pcDNA3.1–3 × Flag vector, and this construct was named SALL4-TrM.

293FT cells were grown in Dulbecco’s Modified Eagle Medium (DMEM; C11995500BT, Gibco, USA) supplemented with 10% fetal bovine serum (Gibco, USA), GlutaMAX™ supplement (100 × ; 35,050–061, Gibco, USA), MEM non-essential amino acid solution (NEAA, 100 × ; 11,140–050, Gibco, USA), and Penicillin/Streptomycin (15,140–122; Gibco, USA) under 5% CO_2_ conditions.

Transient transfection was performed in 12-well plates when the cells were approximately 30% confluent, using the jetPRIME transfection reagent (101,000,046, Polyplus, Illkirch, France) according to the manufacturer’s instructions.

### Immunofluorescent staining

Immunofluorescent staining was performed as previously described [16]. Specifically, an anti-FLAG (DYKDDDDK Tag) mouse primary antibody (#8146; Cell Signaling Technology, Danvers, MA, USA) was used at a dilution of 1:1000. Alexa Fluro 488 conjugate anti-mouse IgG (H + L), F(ab’)2 Fragment secondary antibody (ZB-0512, ZSBIO, Beijing, China) was used at a dilution of 1:100.

### RNA-sequencing and data analysis

RNA sequencing was performed by Berry Genomics (Beijing, China). RNA quality was analyzed using an Agilent 2100 Bioanalyzer (Agilent Technologies, Palo Alto, CA, USA). cDNA libraries were constructed using the Illumina NovaSeq 6000 sequencing platform (San Diego, CA, USA). Samples from three biological replicates were analyzed. Eukaryotic mRNA was enriched using magnetic beads with Oligo (dT). The mRNA was then fragmented into short segments, and the first strand of cDNA was synthesized using the segmented mRNA as a template. The second cDNA strand was synthesized by adding buffer solution, dNTPs, and enzymes. The obtained double-stranded cDNA was purified, poly-A tails were added, fragments were selected, and cDNA libraries were enriched. A Qubit 3.0 fluorimeter was used for preliminary quantification, and qPCR was used for accurate quantification. The reads were filtered to obtain clean, high-quality reads for gene expression and structural analyses. Genes that showed a > twofold difference (FC > 2) and *p* < 0.05 were selected for further analysis. Gene Ontology (GO) analysis was performed using the gene annotation and analysis resource Metascape (http://metascape.org/gp/ index.html).

### Realtime quantitative PCR

Total RNA extraction, cDNA synthesis, and quantitative PCR (q-PCR) were performed as previously described [17]. Relative gene expression levels were normalized to the critical threshold value of the housekeeping gene *ACTB*. The primers used for all sequences are listed in Supplementary Table 1. Real-time PCR was performed in triplicate.

### Western blotting

Preparation of cell lysates, quantification of protein concentration, and western blotting have been described in a previous study [17]. Antibodies against the DYKDDDDK Tag (FLAG, 9A3), mouse monoclonal antibody (#8146, Cell Signaling Technology, MA, USA), WNT11 polyclonal antibody (ab96730, Abcam), PAX2 polyclonal antibody (ab79389, Abcam), and GAPDH monoclonal antibody (60,004-1-Ig; Proteintech) were used.

## Results

### WES analysis of the fetus with aplasia of the radii and unilateral renal agenesis

**Fig. 1:**
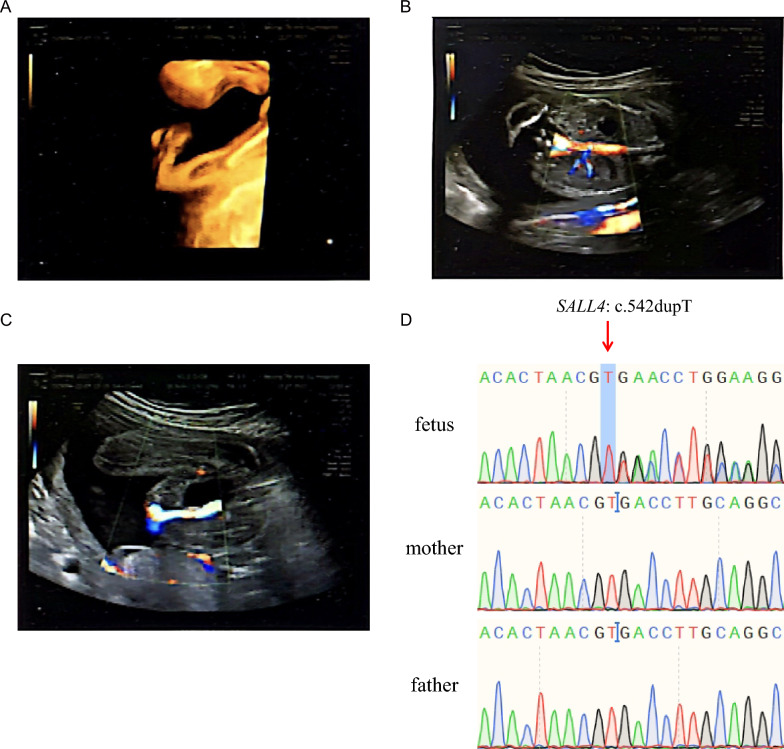
*SALL4* variant in the fetus. **A** Absence of radius. **B** Left renal agenesis and right kidney duplication. **C** Single umbilical artery. **D** The fetus carried the *SALL4* variant c.542dupT, but neither the mother nor the father carried it, indicating that the c.542dupT was a de novo variant

A pregnant woman underwent prenatal examination, and ultrasound showed fetal presence of radii aplasia (Fig. 1A), left renal agenesis, right kidney duplication (Fig. 1B), and a single umbilical artery (Fig. 1C). WES was performed on samples from the fetus, pregnant woman, and her husband. Variants with minor allele frequencies greater than 0.001 in databases such as 1000G, dbSNP, ESP6500, and gnomAD were filtered out. Since the abnormalities described only occurred in the fetus and not in the pregnant woman or her spouse, we analyzed this family based on a de novo mutation pattern. This result was consistent with our hypothesis. We identified a novel *SALL4* variant, c.542dupT, in fetal WES data; however, this variant did not exist in the pregnant woman and her spouse. Sanger sequencing confirmed that the fetus harbored the c.542dupT heterozygous variant, while neither the pregnant woman nor her spouse carried this variant (Fig. 1D). c.542dupT is an extremely rare variant, not found in the gnomAD database. It is predicted to translate into the p.Thr182Aspfs*204 protein and is classified as a likely pathogenic variant according to the American College of Medical Genetics and Genomics (ACMG) classification.

### SALL4 truncated protein mis-localized in the cytoplasm

**Fig. 2:**
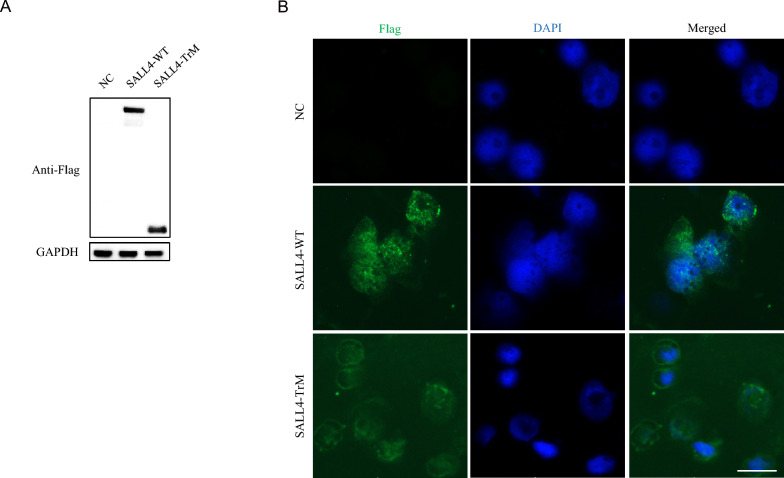
Mis-localization of the SALL4 truncating protein. **A** Western Blot showing that the wild type SALL4 (SALL4-WT) protein and SALL4 truncating (SALL-TrM) protein could be effectively expressed in cells. SALL4-WT and SALLT-TrM were both linked with Flag tag, and ACTB served as an internal reference protein. **B** Localization of SALL4 protein in cells. Immunofluorescence staining showing SALL4-WT mainly localized in the nucleus and SALL4-TrM mainly localized in the cytoplasm. DAPI was used for nucleus staining. Scale bar = 50 μm

To study the cellular and molecular effects of the SALL4 truncated protein, we constructed a shortened protein that retained the N-terminal 181 amino acids of SALL4, fused to a C-terminal Flag tag. We transfected cells with the wild-type plasmid SALL4-WT-Flag and the truncated plasmid SALL4-TrM-Flag and found that both plasmids effectively expressed their proteins in the cells (Fig. 2A). Immunofluorescence staining showed that SALL4-WT protein was mainly localized in the cell nucleus, whereas the truncated protein was mainly localized in the cytoplasm (Fig. 2B).

### SALL4 transcriptional activity was compromised in the SALL4 truncated protein

**Fig. 3:**
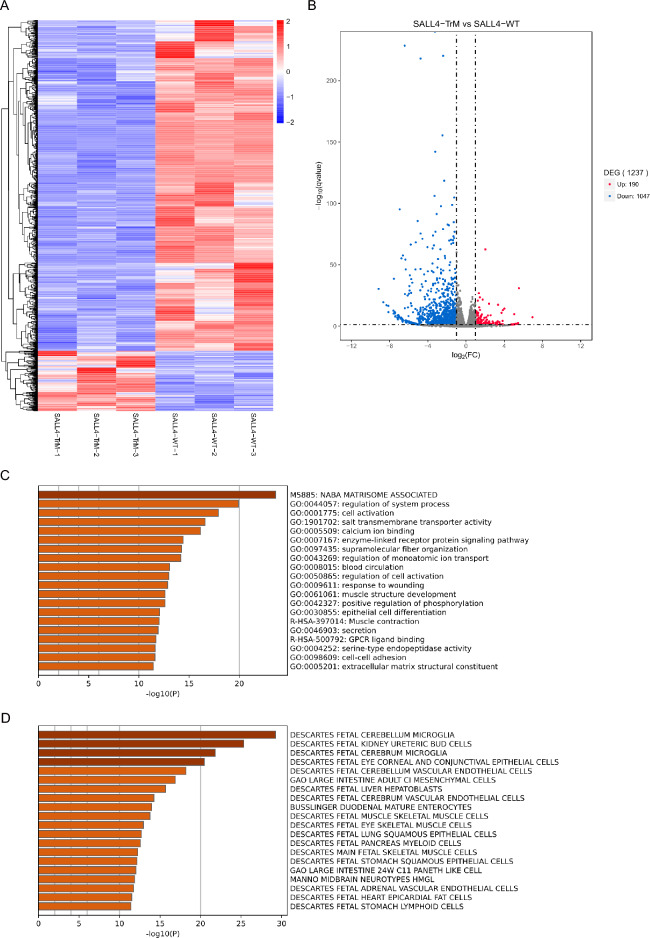
RNA-sequencing analysis of SALL4-WT- and SALL4-TrM- expressing cells. **A** Heatmap illustration shows total differentially expressed genes (DEGs) in cells expressing SALL4-WT and SALL4-TrM. Three biological replicates for each group. Red represents high gene expression and blue represents low gene expression. **B** Volcano plot displays DEGs (upregulated, red; downregulated, blue) in cells expressing SALL4-TrM compared to cells expressing SALL4-WT. FC, fold change. **C** Enriched biological processes in Gene Ontology (GO) analysis of the 1,047 downregulated genes in cells expressing SALL4-TrM compared to those expressing SALL4-WT. **D** Summary of enrichment analysis in Cell Type Signatures. Enriched Cell Type Signatures in Gene Ontology (GO) analysis of the 1,047 down-regulated genes in cells expressing SALL4-TrM compared to those expressing SALL4-WT

The SALL4 truncated protein was mainly localized in the cytoplasm, suggesting that its function as a transcription factor may be impaired. Therefore, we used RNA sequencing to investigate whether the SALL4 truncated protein has a transcriptional activation ability similar to that of wild-type SALL4. Heatmap analysis of the RNA sequencing results showed that the transcriptional profile of cells expressing SALL4-TrM was significantly altered compared with that of cells expressing SALL4-WT (Fig. 3A). The volcano plot shows the number of upregulated and downregulated genes in the SALL4-TrM group relative to those in the SALL4-WT group (Fig. 3B). These results indicated that SALL4-TrM was unable to activate the expression of downstream genes as effectively as SALL4-WT. GO analysis showed that many biological processes were enriched among the 1,047 downregulated genes, such as cell activation, salt transmembrane transporter activity, and calcium ion binding (Fig. 3C). We sought to determine the cells in which these differentially expressed genes were enriched. We found that fetal ureteric bud cells were a cell type with significant enrichment (Fig. 3D), indicating that these differentially expressed genes are crucial for the development of fetal ureteric bud cells, which is consistent with the phenotype of patients harboring *SALL4* mutations.

### WNT11 and PAX2, key regulators of kidney development, were significantly lower expressed in the SALL4-TrM group

**Fig. 4:**
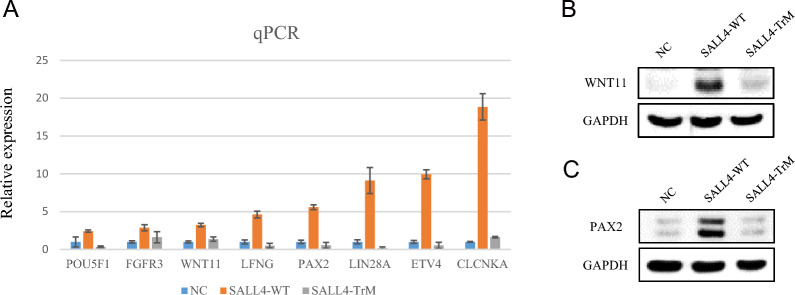
SALL4 transcriptionally activates *WNT11* and *PAX2* expression. **A** Validation of RNA-seq data using quantitative RT-PCR in cells expressing SALL4-WT, SALL4-TrM, and control plasmids. NC, negative control. Three biological replicates for each group. **B** Western blot analysis of WNT11, with GAPDH as the internal reference protein. **C** Western blot analysis of PAX2, with GAPDH as the internal reference protein

We validated the differentially expressed genes identified through RNA-seq using qPCR and found that SALL4-WT activated the expression of *POU5F1*, *FGFR3*, *WNT11*, *LFNG*, *PAX2*, *LIN28A*, *ETV4,* and *CLCNKA*, whereas SALL4-TrM did not (Fig. 4A). Wnt11 plays a role in the earliest stages of kidney development; specifically, it is expressed at the tips of the branching ureteric epithelium [18–20]. Mutations in the *Wnt11* gene lead to defects in ureteric branching morphogenesis, resulting in kidney hypoplasia in mice [18]. The expression of *Wnt11* is positively regulated by Gdnf and functions downstream of the RET/GFRA1 signaling pathway [18, 21]. *PAX2* is essential for fetal kidney and ureter development and mutations in this gene can cause congenital abnormalities of the kidney and urinary tract (CAKUT) [12, 22, 23]. We then focused on the changes in the expression of *WNT11* and *PAX2* in SALL4-WT or SALL4-TrM expressing cells. Western blot analysis revealed that SALL4-WT could significantly activate the expression of WNT11 and PAX2, but SALL4-TrM failed (Fig. 4B and C). Therefore, we proposed that SALL4 may play a role upstream of WNT11 and PAX2 during fetal kidney development by transcriptionally activating the expression of *WNT11* and *PAX2*.

## Discussion

In this study, we performed WES on a fetus with renal and skeletal developmental abnormalities and identified a frameshift mutation in *SALL4*. To demonstrate that this mutation was the cause of the fetal anomaly, we constructed a mutant expression vector and expressed the mutant protein in cells, finding that the SALL4 mutant protein was mislocalized from the nucleus to the cytoplasm. Furthermore, RNA sequencing analysis revealed that the SALL4 mutant protein significantly lost its transcriptional activation ability, affecting downstream genes that were mainly enriched in fetal kidney ureteric bud cells, suggesting that the SALL4 mutant protein may affect the development and function of fetal kidney ureteric bud cells. A more in-depth mechanistic analysis revealed that SALL4 regulates the expression of the downstream genes *WNT11* and *PAX2*, both of which have been shown to play important roles in kidney development. Therefore, the SALL4 mutant protein may cause renal agenesis by failing to transcriptionally activate *WNT11* and *PAX2*, thus affecting kidney development.

Previous studies have found that heterozygous variants in *SALL4* are associated with CAKUT or renal agenesis, with reported variants including p.Arg329His, p.Ser579Arg, p.Arg831Gln, p.Val837Gly, and p.Lys1049Arg [12, 24–27]. However, these studies did not experimentally confirm a causal relationship between these variants and the disease. In this study, we discovered that mutant SALL4 proteins are mislocalized to the cytoplasm. When SALL4 mutations result in its mislocalization to the cytoplasm, the protein may lose its normal regulatory functions in the nucleus, potentially leading to various cellular dysfunctions. SALL4 is normally involved in the regulation of gene expression, which is critical for development and stem cell maintenance. Mislocalization can lead to the loss of regulatory functions and may affect cell differentiation, proliferation, and apoptosis pathways. Moreover, our study further found through RNA sequencing that the mutant SALL4 proteins failed to function as transcription factors, as demonstrated by the inability to activate the transcription of most downstream genes that wild-type SALL4 could regulate.

RNA sequencing data also suggested that the differentially expressed genes were mainly enriched in fetal kidney ureteric bud cells, which are specialized cells found in the developing kidney. These cells originate during the early stages of embryonic kidney development. During embryogenesis, the kidney forms a series of structures known as nephrons, which include ureteric buds. These buds arise from the embryo and subsequently differentiate into various cell types, including fetal kidney ureteric bud cells [28]. These cells play crucial roles in kidney development by contributing to the formation of the collecting duct system, which helps regulate fluid balance and filter waste products from the blood. Additionally, fetal kidney ureteric bud cells are involved in branching morphogenesis, a process essential for the proper growth and organization of the kidney’s tubular network [29]. Overall, they are vital for the structural integrity and functional capacity of developing kidneys.

## Conclusions

In summary, this study identified a *SALL4* mutation that leads to renal agenesis and investigated the molecular mechanisms underlying its pathogenicity. This mutation may disrupt the transcription of *WNT11* and *PAX2*, thereby affecting the development and function of fetal kidney ureteric bud cells, ultimately resulting in renal agenesis. This study elucidates the molecular mechanism by which *SALL4* mutations cause renal agenesis and provides new targets for the treatment and intervention of this disease.

## Supplementary Information

Supplementary file1

Supplementary file2

## Data Availability

The variation data reported in this study have been deposited in the Genome Variation Map (GVM) [30] at the National Genomics Data Center, Beijing Institute of Genomics, Chinese Academy of Sciences, and China National Center for Bioinformation [31] under the accession number GVM000715 (https://ngdc.cncb.ac.cn/gvm/getProjectDetail?Project=GVM000715).
