# Supplementary material for: A de novo SALL4 mutation causes unilateral renal agenesis by misregulating genes involved in kidney development
Source: Orphanet J Rare Dis. 2025 Jun 7;20:289. doi: 10.1186/s13023-025-03833-x (PMC12145589; doi:10.1186/s13023-025-03833-x)
Supplement: Supplementary file 1 — Supplementary file1 [file 13023_2025_3833_MOESM1_ESM.pptx]

## Slide 1
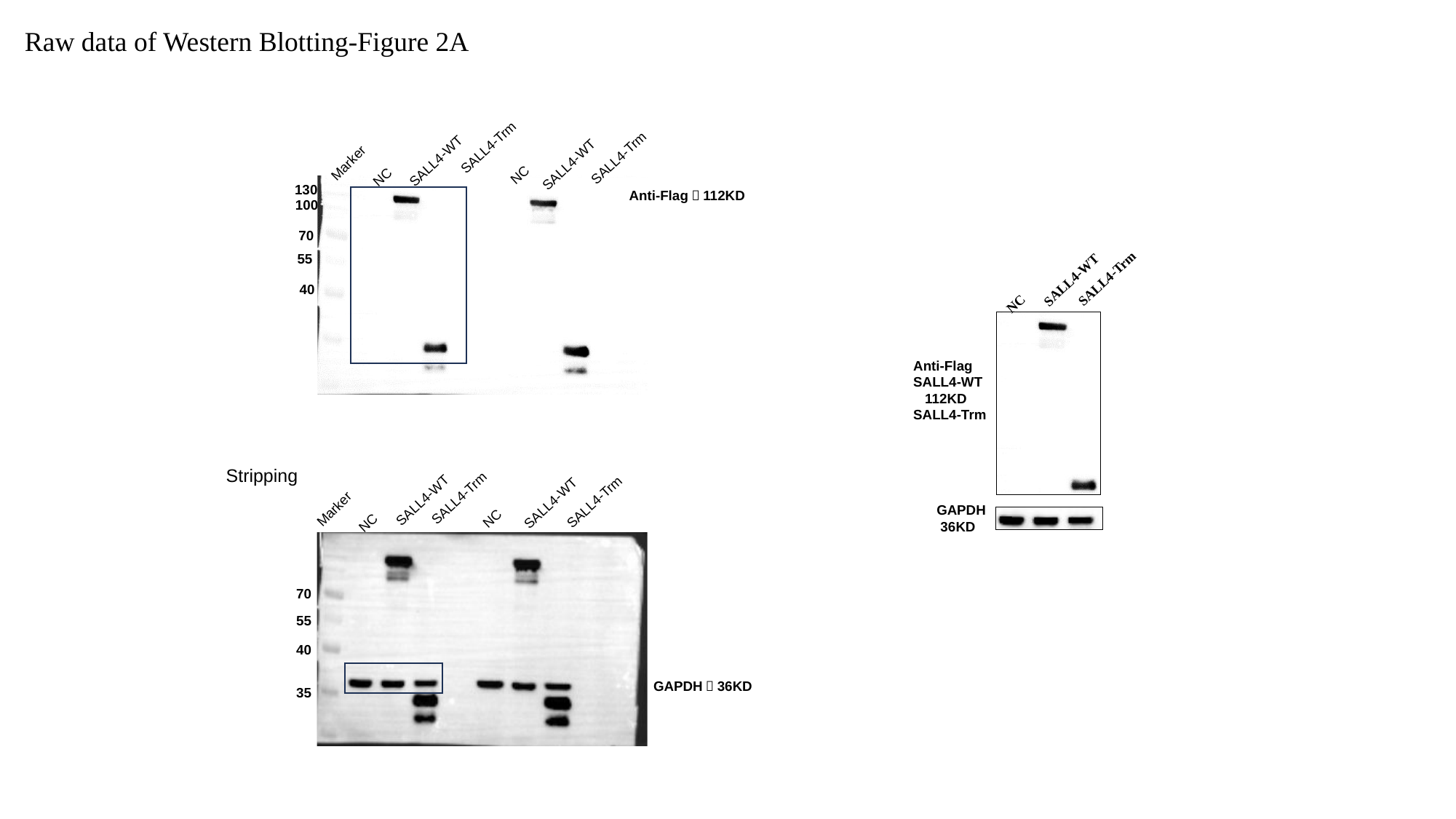

Raw data of Western Blotting-Figure 2A
SALL4-Trm
SALL4-WT
Marker
SALL4-WT
SALL4-Trm
NC
NC
130
Anti-Flag：112KD
100
70
55
SALL4-WT
SALL4-Trm
40
NC
Anti-Flag
SALL4-WT
 112KD
SALL4-Trm
Stripping
SALL4-WT
SALL4-WT
Marker
SALL4-Trm
SALL4-Trm
NC
GAPDH
 36KD
NC
70
55
40
GAPDH：36KD
35

## Slide 2
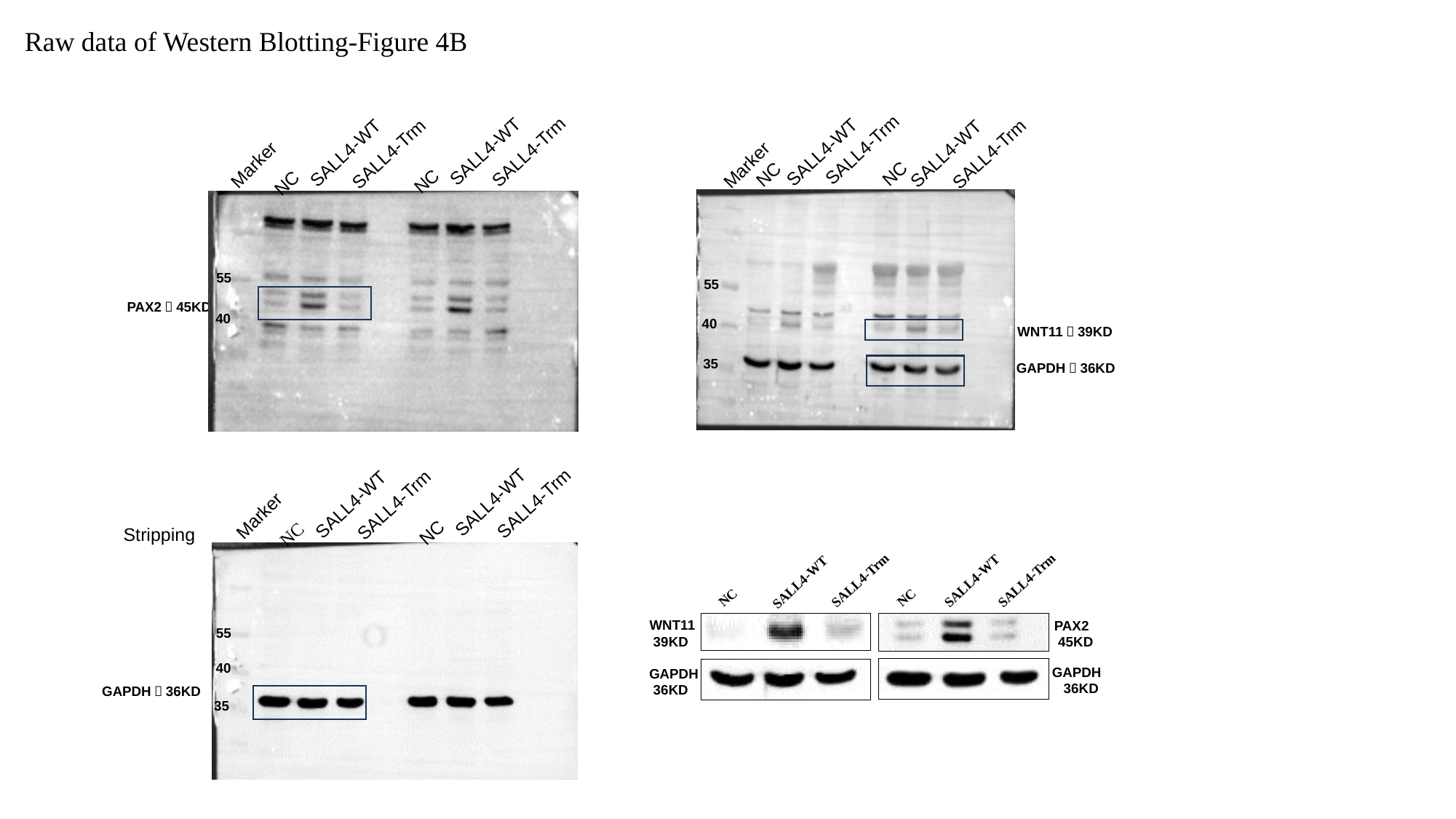

Raw data of Western Blotting-Figure 4B
SALL4-Trm
SALL4-WT
SALL4-WT
SALL4-WT
SALL4-WT
SALL4-Trm
SALL4-Trm
SALL4-Trm
Marker
Marker
NC
NC
NC
NC
55
40
WNT11：39KD
35
GAPDH：36KD
55
PAX2：45KD
40
SALL4-WT
SALL4-WT
SALL4-Trm
SALL4-Trm
Marker
NC
NC
Stripping
SALL4-WT
SALL4-WT
SALL4-Trm
SALL4-Trm
NC
NC
WNT11
 39KD
PAX2
 45KD
55
40
GAPDH
 36KD
GAPDH
 36KD
GAPDH：36KD
35
