# Supplementary material for: A de novo SALL4 mutation causes unilateral renal agenesis by misregulating genes involved in kidney development
Source: Orphanet J Rare Dis. 2025 Jun 7;20:289. doi: 10.1186/s13023-025-03833-x (PMC12145589; doi:10.1186/s13023-025-03833-x)
Supplement: Supplementary file 2 — Supplementary file2 [file 13023_2025_3833_MOESM2_ESM.docx]

**Supplementary Table 1.** DNA Primers used in this study.

| **Gene** | **Gene Accession Number** | **Forward Primer** | **Reversed Primer** | **Experiment** |
| --- | --- | --- | --- | --- |
| *POU5F1* | NM_002701.6 | AGTGAGAGGCAACCTGGAGA | GTGAAGTGAGGGCTCCCATA | qPCR |
| *FGFR3* | NM_000142.5 | CCCAAATGGGAGCTGTCTCG | CCCGGTCCTTGTCAATGCC | qPCR |
| *WNT11* | NM_004626.3 | GACCTCAAGACCCGATACCTG | TAGACGAGTTCCGAGTCCTTC | qPCR |
| *LFNG* | NM_002304.3 | GTCAGCGAGAACAAGGTGC | GATCCGCTCAGCCGTATTCAT | qPCR |
| *PAX2* | NM_000278.5 | TCAAGTCGAGTCTATCTGCATCC | CATGTCACGACCAGTCACAAC | qPCR |
| *LIN28A* | NM_024674.6 | TGCGGGCATCTGTAAGTGG | GGAACCCTTCCATGTGCAG | qPCR |
| *ETV4* | NM_001986.4 | CAGTGCCTTTACTCCAGTGCC | CTCAGGAAATTCCGTTGCTCT | qPCR |
| *CLCNKA* | NM_004070.4 | ACCCTGACGCTATTCTCAGAG | CGATGTCACGAAGAGGGACTG | qPCR |
| *ACTB* | NM_001101.5 | GCACAGAGCCTCGCCTT | GTTGTCGACGACGAGCG | qPCR |
